# On the use of cartographic projections in visualizing phylo-genetic tree space

**DOI:** 10.1186/1748-7188-5-26

**Published:** 2010-06-08

**Authors:** Kenneth Sundberg, Mark Clement, Quinn Snell

**Affiliations:** 1 Computer Science Department, Brigham Young UniversityProvo, UT 84602, USA

## Abstract

Phylogenetic analysis is becoming an increasingly important tool for biological research. Applications include epidemiological studies, drug development, and evolutionary analysis. Phylogenetic search is a known NP-Hard problem. The size of the data sets which can be analyzed is limited by the exponential growth in the number of trees that must be considered as the problem size increases. A better understanding of the problem space could lead to better methods, which in turn could lead to the feasible analysis of more data sets. We present a definition of phylogenetic tree space and a visualization of this space that shows significant exploitable structure. This structure can be used to develop search methods capable of handling much larger data sets.

## Background

Phylogenetic analysis has become an integral part of many biological research programmes. These include such diverse areas as human epidemiology [1,2], viral transmission [3,4], and biogeography [5]. With the advent of new automated sequencing techniques, the ability to generate data for inferring evolutionary histories (phylogenies) for a great diversity of organisms has increased dramatically. Researchers are now commonly generating many sequences from many individuals. However, our ability to analyze the data has not kept pace with data generation.

Phylogenetic search is a difficult problem. When parsimony is used as the optimality criterion the problem is known to be NP-complete [6]. The search problem itself, independent of scoring, is known to be NP-Hard [7]. This means that optimal phylogenetic searches on even hundreds of taxa will take years to complete and heuristic searches for near optimal trees must be used.

A variety of heuristic search methods have been used to find optimal trees within a tree space. The most common method is to search tree space using tree rearrangements [8-11]. Other methods such as those based on Bayesian inference [12], or genetic algorithms [13] also exist. However all of these methods rely only on local information to guide the phylogenetic search. This limitation arises because no global exploitable structures have been previously observed in tree space.

Greater understanding of the problem space may allow more sophisticated search techniques to be applied, with a consequent improvement in the effectiveness of the search. One technique that can be used to better understand the space of phylogenetic search, and the behavior of search algorithms within this space, is visualization. This includes two separate activities: first, defining the search space of phylogenetic trees, or tree space, and second, developing methods to display tree space in a way that is exploitable in search techniques.

This visualization must have the following properties to be useful.

• Each tree should map to a single deterministic position. Otherwise the method is restricted to post-processing, and cannot be used to guide a search.

• Distance between trees should be easy to calculate. If it is not, the visualization will not be able to be used in real time to guide a search.

• The visualization should reveal exploitable structure. This is important because if a visualization shows no structure it provides no guidance for a search.

• This mapping should be reversible, meaning that there should be a method of turning a position into a tree. This is necessary, as to be useful in searching it must be possible to quickly find trees in the space suggested by the visualization.

This work presents an elegant linear projection of trees. This projection can be computed much faster than current alternatives and is better at preserving structural continuity between trees after the projection. Furthermore this projection is deterministic, allowing it to be used as an inline rather than a post-process analysis. This property coupled with the structural preservation allows the consideration of novel search strategies in the new projected space.

The Results and Discussion section presents a definition of tree space and an elegant projection of that space that has all four of these desirable properties. This projection is then used to visualize the tree space and expose structure that can be exploited to guide the searches of common, but computationally expensive, methods.

## Related Work

**Figure 1:**
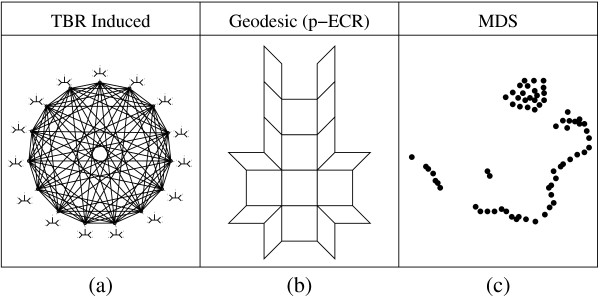
**A visual comparison of three tree spaces previously used**. The graph structure induced by TBR (a) moves is highly connected. The geodesic structure (b) consists of tiles of Euclidean space (orthants) each consisting of one topology with all its possible branch lengths. These tiles are joined together along their edges in accordance with valid p-ECR moves. Finally Multidimensional Scaling (MDS) (c) plots trees in locations that preserves some distance metric. A typical search is shown where a long tail of trees is followed by a larger group of topologically similar trees.

Tree space consists of all of the possible phylogenetic trees for a given set of taxa and their relationships with each other. This space is the domain of whatever search strategy is employed. Previous search strategies have not explicitly defined this domain, and the tree space that implicitly arises from these strategies is very cumbersome to work with. Tree spaces have also been explicitly defined without designing algorithms to take advantage of these spaces. This is primarily due to a lack of exploitable structure in these explicitly defined tree spaces. Figure 1 contains a visual comparison of three tree spaces that have been used previously and are discussed in the following sections.

### Subtree Transfer Induced Spaces

The most common tree spaces used in phylogenetic search are the spaces implicitly defined by the subtree transfer operations, such as Tree Bisection and Reconnection (TBR) or Subtree Prune and Regraft (SPR), used during the search. These operations in turn induce distances between trees [14]. These tree spaces take the form of graphs where each node is a specific tree. Each pair of trees that can reach each other with a single subtree transfer operation is connected with an edge of the graph.

This type of space is very amenable to hill climbing, a search strategy in which the search moves from a tree to its best neighboring tree until no neighbor trees are better than the current tree. The typical phylogenetic search begins at some node in this graph of tree space corresponding to an initial tree. This tree is typically either selected randomly, determined by the user, or is built using a heuristic. Common choices for this heuristic include UPGMA and stepwise maximum parsimony. The tree is then modified using a subtree transfer operation such as Nearest Neighbor Interchange (NNI), Subtree Prune and Regraft (SPR), Tree Bisection and Reconnection (TBR), or p-Edge Contraction and Refinement (p-ECR) [15]. The new best node becomes the starting node and the process is repeated until convergence. This is also the space used by Keith *et al*. [16] to build their generalized Gibbs sampler.

**Figure 2:**
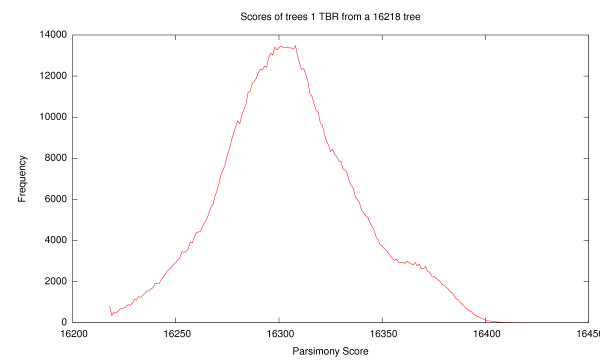
**The frequency of various parsimony scores for trees found within 1 TBR rearrangement of a tree with a score of 16,218, the best known score on the Zilla data set**. Note the wide spread of scores and that most neighbor trees are much worse than the initial tree.

Unfortunately, though this space has been commonly used for searching, it is not easily visualized. For example using TBR, a very popular subtree transfer operation, the graph that represents this tree space has *O*(*n*!!) nodes and each node is degree *O*(*n*^3^). Displaying this graph is clearly not practical for any problem of significant size. Worse, as this tree space is essentially a graph, there is no significant meaning to position, violating the first two criteria for a useful visualization. Also, distance can be extremely difficult to compute. Calculating TBR distance is NP-Hard [14]. These difficulties violate the third criterion. Finally this graph structure shown in Figure 1a does not exhibit exploitable structure, the fourth criterion, as trees of similar score are not grouped together. As shown in Figure 2, the quality of trees that are within 1 TBR rearrangement of a given tree varies wildly over the range of possible scores. Furthermore, due to the graph structure of the space there is no way to distinguish one such tree from another, without performing the rearrangement and examining the resulting tree.

### Geodesic Tree Space

Billera *et al*. [17] introduced a new description of tree space, which has been further refined by Hultman [18]. Under this description, each fully resolved (bifurcating) topology is given its own orthant, the higher dimensional analog of a graph quadrant. Each dimension of the orthant corresponds to one of the branches in the topology, and the value associated with that dimension is the length of that branch. Within each orthant, distance is a simple Euclidean distance. At the edges of the orthant, where at least one coordinate becomes zero, the tree becomes an unresolved (multifurcating) tree. This unresolved tree has a corresponding point on each of the orthants that represent a potential resolution of this tree. The distance between these points on separate orthants is defined to be zero, thus forming a geodesic space. These connections between orthants are directly related to p-ECR rearrangements. The structure of this space can be seen in Figure 1b.

This space is unlike the tree space induced by subtree transfer operations. The branch lengths of the trees are included and this tree space is continuous. However, because it is a geodesic, it can be difficult to calculate distances, though recent work [19-21] has begun to address this issue. Unfortunately, like the subtree transfer induced spaces used during phylogenetic search, geodesic tree space is not easily visualized due both to the high dimensionality of each orthant and the complex connections between orthants. These connections are based on a subtree transfer operation, p-ECR, and so like the tree space defined by TBR there is no significant meaning to position between orthants. Thus, like the TBR induced tree space, this tree space does not meet the criteria for a good visualization. While position and trees are tightly connected, distance is difficult to compute and it is not clear that there is any exploitable structure.

### Multidimensional Scaling

Multidimensional Scaling (MDS) has also been used to visualize tree space [22,23]. This method does not directly define a tree space, rather it uses the space induced by the distance metric used for the MDS. In both the work of Hillis *et al*. [23] and the prior work by Amenta and Kilinger [22], Robinson-Foulds distance was used. This distance is a measure of how many branches are not in common between two trees. MDS is a highly non-linear projection, as it moves points around to minimize the sum of the squared differences of the distances between points before and after the projection. One difficulty is that in the presence of many points, clusters formed by MDS may not reflect topological similarity, but instead reflect the best found compromise in this strain function.

Using this method Hillis *et al*. [23] were able to show some important characteristics of phylogenetic search. The most notable characteristic visualized was the presence of plateaus, large groups of closely related trees, that tend to slow down the search.

There are however some significant limitations to their use of MDS, which may not apply to the many variants of Multidimensional Scaling [24] or to other manifold-based methods. First, MDS is strictly a post-processing step. All of the points to be projected must be known beforehand, which limits the method to analysis of a search. Secondly there is no meaning to the space between points. It is not possible under MDS to determine a tree that would map to a specific point. Third, the axes of the new space have no consistent meaning. The only thing that MDS tries to preserve is some sense of distance; direction does not have any meaning after MDS is performed. As a result of these limitations, while MDS is a good visualization technique it does not meet the criteria of this work. This is primarily due to the highly non-linear and irreversible nature of the MDS transformation. MDS can be a very descriptive visualization, but it is a poor predictive visualization.

## Results and Discussion

Another tree space is one defined in terms of partitions of taxa. A projection can be defined from this space which both deterministically maps trees to single points and is reversible. These properties give us the first three criteria for a good tree space and visualization. In the results section we show that this space also displays exploitable structure.

There are several varieties of trees that can be used in phylogenetics. Since only one specific set of n taxa will be considered at any time we constrain tree space to contain only trees of exactly those *n *taxa. Both candidate scoring metrics (likelihood and parsimony) work with unrooted trees so the space is further constrained to contain only unrooted and fully resolved trees.

**Definition 1. **An *n*-tree is a graph in which all vertices have degree one or three, with exactly *n *vertices of degree one.

Every branch in an *n*-tree divides the taxa on the tree into two sets, one on each side of the branch. Thus every branch can be thought of as a partition of the taxa. Some of these branches, those that connect to the leaves, are common to all *n*-trees. These branches are not useful in discriminating between different tree topologies and so are called trivial.

**Definition 2. **A trivial branch is a branch that connects a leaf node with an internal node.

Given *n *taxa there are 2^*n*-1 ^- *n *- 1 possible nontrivial partitions of those taxa. We define a space, called *split space*, where every possible nontrivial partition is associated with a unique dimension. We denote the split space associated with trees of *n *taxa as .

The location of a given tree in  is a vector, where each element of the vector is 0 if the corresponding partition is not part of the tree and 1 if the partition is present in the tree. There is a one-to-one mapping between vectors in split space and *n*-trees.

The mapping from an *n*-tree to a vector in  is simple. Initially, every element of the vector is set to 0. A non-trivial branch is selected and the associated partition is created by putting all taxa on one side of the branch into the first group in the partition and all other taxa in the second. The element in the vector associated with this partition is set to 1. This process is repeated for each non-trivial branch. This mapping is one-to-one but not onto, as there are more possible vectors than *n*-trees. This is because there exist conflicting partitions which cannot both be in one tree; however there are vectors which would include these conflicts.

**Figure 3:**
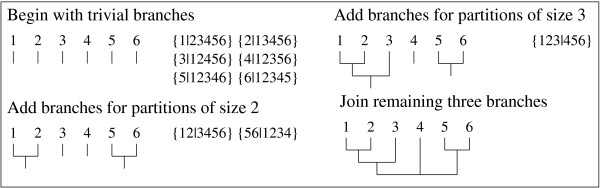
**Converting a six taxon partition set to an unrooted tree structure**.

Building an *n*-tree from a vector in  is also possible. However given a vector that does correspond to a valid tree, that tree can be reconstructed in the following manner. This is very similar to the method proposed by Gusfield [25]. First, all of the trivial branches are added to the tree. Next, all non-trivial partitions where the smaller group contains two taxa are considered. Each of the two taxa in the smaller group are joined at a new internal node and a new branch is added to that node. Next, partitions with incrementally larger small groups are considered, and their subclades which have already been built are joined at new internal nodes. After all non-trivial partitions have been considered, there will remain three clades. These three subtrees are joined together at the final internal node and the tree has been reconstructed. Figure 3 graphically shows this reconstruction. As there is a mapping from an *n*-tree to a vector in  and the reverse mapping also exists, these trees and vectors are equivalent.

## The Hypersphere of Trees

A hypersphere consists of the set of all points which are equidistant from a given center point. It is the higher dimensional analog of circles and spheres. The set of all vectors in  which correspond to valid *n*-trees has this structure as shown in Theorem 4.

**Lemma 3. ***All n-trees have *2*n *- 3 *branches, and n - *3 *of which are nontrivial.*

*Proof*. See Waterman [26], Proposition 14.1

**Theorem 4. ***All n-trees lie on a hypersphere in *.

*Proof*. By Definition 1, *n*-trees are fully resolved. All fully resolved trees on *n *taxa have *n *- 3 nontrivial branches by Lemma 3. As each such branch corresponds to exactly one of the possible partitions, an arbitrary *n*-tree in  will have exactly *n *- 3 axes along which the coordinate of the tree will be 1 and all other axes will have a coordinate of 0. The Euclidean distance to this point from the origin of  will therefore be , which is the same for all *n*-trees. As all *n*-trees are equidistant from the origin, they lie on a hypersphere.

### Projecting the Sphere

Directly visualizing the hypersphere model is clearly infeasible as the number of dimensions that would need to be included quickly exceeds the number of dimensions that we can conveniently visualize. Therefore some form of dimension reduction is needed.

### Sphere to Plane Projections

**Figure 4:**
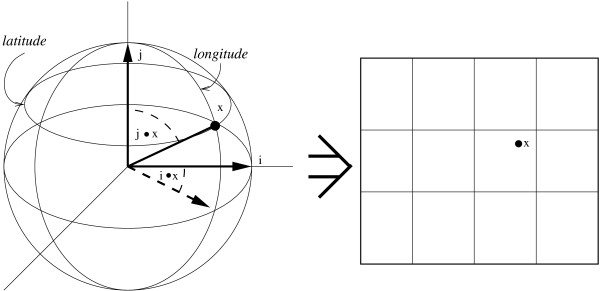
**Cartographic projection of a sphere onto a plane, the most familiar of which is used in map making**. Two vectors are selected, indicated as *i *and *j*. For any given point *x *on the sphere the inner products *i *• *x *and *j *• *x *are computed. These two quantities become the new coordinates of the point on the map.

Cartographic projections [27] are particularly apt at sphere to plane transformations. The basic cartographic projection takes a hypersphere in *n *dimensions and projects it onto a hyperplane of *n *- 1 dimensions. This is done by selecting *n *- 1 vectors, typically chosen from a basis set. Figure 4 shows how this reduction can project three dimensional data onto two dimensions. The inner product of each point on the hypersphere to be projected with each of the selected vectors is computed. These inner products become the coordinates of the projected point on a hyperplane of *n *- 1 dimensions.

This cartographic projection can be extended to a new projection that reduces the dimensionality of the space more than the basic cartographic projection. Reducing the n dimensional space by one dimension when n grows as the number of possible partition sets is not significant. Therefore, rather than choosing *n *- 1 vectors which results in a *n *- 1 dimensional space, three vectors are used, yielding a three dimensional space. Three dimensions are used because it is well known how to display 3-D data, and the use of three dimensions preserves more structure than if the data were reduced to two dimensions.

**Figure 5:**
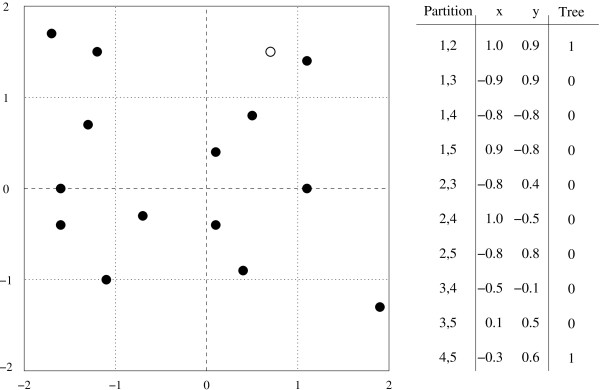
**A 2-D cartographic projection of all 5 taxon trees, with reference vectors**. The vector for the tree ((1,2),3,(4,5)) is also shown. The point corresponding to this tree is highlighted in the graph.

As an example of this process, consider all trees with five taxa numbered 1-5 respectively. Every non-trivial branch has two taxa on one side and three on the other. There are ten such partitions, yielding a ten dimensional space. To project this space onto a two dimensional plane, two reference vectors are required. The vectors chosen, along with the projected positions of all five taxon trees are shown in Figure 5. In these examples trees are expressed in Newick format, with the taxa represented by numbers and parenthesis to indicate clades. The tree ((1,2),3,(4,5)) is mapped in the following manner. The partition (1,2) has an *x *value of 1.0 and a *y *value of 0.9. Likewise the partition (4,5) has an *x *value of -0.3 and a *y *value of 0.6. These values are added together to give the final location of the tree ((1,2),3,(4,5)) at the point (0.7,1.5).

The spherical structure of trees in  shown in Theorem 4, permits the use of cartographic projections. As this class of projections is deterministic, the position of a tree after cartographic projection is deterministic and depends only on the tree in question, thus satisfying the first visualization criterion. Furthermore the space both before and after the projection is a simple Euclidean space where distance is easily calculated, satisfying the second criterion. The results section shows the exploitable structure revealed by the projection, which satisfies the third criterion. The projection is also reversible, which satisfies the final criterion.

Thus, the hypersphere structure and the use of cartographic projections allow us to represent phylogenetic search in a manner consistent with the original visualization criteria.

## Results

The definitions of  and the cartographic projection are deterministic, reversible and have an easily calculated distance metric, fulfilling three of the four criteria for a useful visualization. The fourth criterion, exploitable structure, is the most important. The cartographic projection places similarly scored trees together in the data sets examined. This creates a gradient, an exploitable structure, which allows future work to develop a gradient descent strategy, which would be an improvement over current hill climbing techniques.

### Locality of Structure

To have any exploitable structure there must be some correlation between position in the projected space and the topology of the trees near that position. Three methods will be considered: first, the method of Cartographic Projections, second, Multidimensional Scaling in two dimensions as in TreeSetVis [23], and finally Multidimensional Scaling in three dimensions to account for any effects from the extra degree of freedom. The test case will be the exaustive set of all trees of seven taxa, with each method running 100 times as they all have random elements. Once each projection is calculated, the nearest *m *neighbors for every tree are found, with *m *ranging from 0 to 25. A majority rule consensus tree is then constructed for each of these neighborhoods. This tree contains only those partitions which are present in a majority of the trees in a neighborhood. The resolution of these trees is reported, with a value of 1 indicating that the tree was fully resolved and a value of 0 indicating that the tree was fully unresolved.

**Figure 6:**
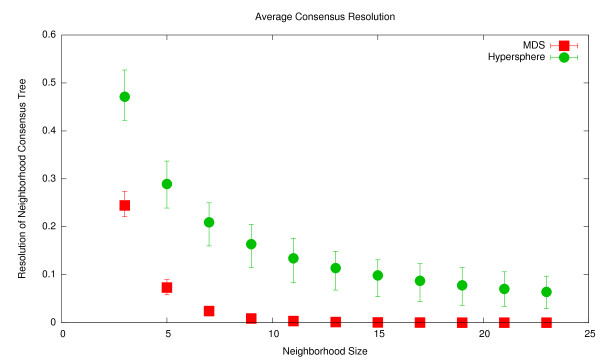
**The average degree of consensus across near neighbors among all trees with 7 taxa, note that higher values are better**. As both cartographic projections and MDS have a random component each point consists of 100 projections with the average, minimum and maximum values for the consensus across all neighborhoods of the given size plotted. MDS was run both in the two dimensional case as in TreeSetVis and in a three dimensional case as the chosen cartographic projection resulted in a three dimensional result.

Figure 6 shows the results of this test. The points are plotted with the minimum, average and maximum values for the resolution. Note that cartographic projections are superior to both two and three dimensional MDS in every case. Not only are close trees more structurally similar, but also the neigborhoods over which some degree of topological similarity is found are much larger. It is thus concluded that cartographic projections produce, in terms of topology, a smoother mapping of tree space. Further this superiority is not due to the added flexibility of projecting onto three dimensions rather than two.

### Results from Nine Taxon Set Exhaustive Searches

To explore the inherent structure of the maximum parsimony problem, several nine taxon data sets out of BAliBASE [28] were fully analyzed. The data set size was selected because with only 135,135 possible solution trees, it was very feasible to exhaustively enumerate all solutions for many different data sets of this size and to plot all of the points. Each set was exhaustively enumerated and scored using PAUP* [10]. The three reference points for the projection were chosen at random. Under this projection each of the possible trees mapped to a unique point in the new three dimensional space. The same projection was used for all of the data sets. These points were then colored according to the parsimony score of the corresponding tree, with white indicating a poor score and black indicating a good score.

**Figure 7:**
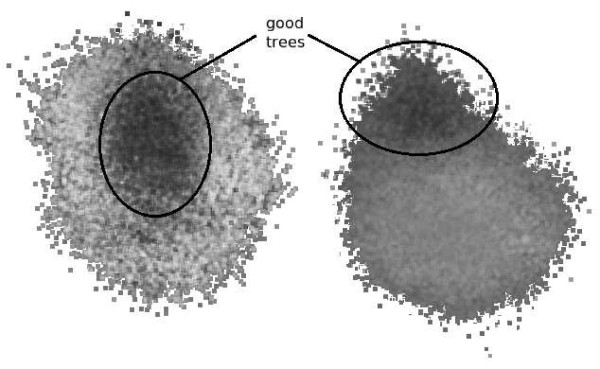
**Two distinct 9 taxon data sets under cartographic projection**. Dark points represent trees with better scores. The set on the left shows clear clustering with good trees near the center of the cloud. The set on the right shows a gradient, with good trees at the upper point of the cloud.

In all of the data sets, there is significant exploitable structure. In some, such as that shown on the right in Figure 7, a clear nearly linear gradient was visible throughout the entire cloud of possible trees. While in others, such as that shown on the left in Figure 7, clustering of scores is clear. Even though the gradient was much more complex, it would still be possible to use gradient descent.

### Visualizing an Exhaustive Search with MDS

**Figure 8:**
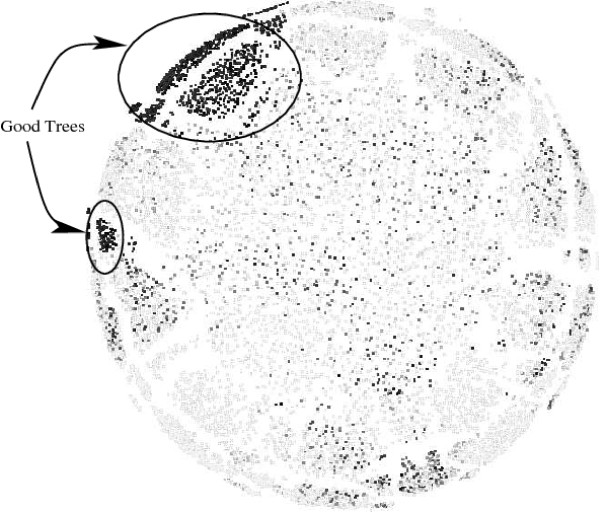
**A multi-dimensional scaling (MDS) visualization of an exhaustive search of 8 taxa**. Dark points represent trees with better scores. Note that there is some clustering of good trees but that they can be found throughout the visualized set.

The tool TreeSetViz was used to produce a visualization of a complete data set for comparison with our cartographic projections. Due to the very high memory requirements of multi-dimensional scaling, it was not possible to use a nine taxon data set. An eight taxon subset was used instead. The program was run overnight to allow the program adequate time to converge to the mapping shown in Figure 8.

A few features are noteworthy. First, the circular shape, which is a result of the hyperspherical nature of tree space. As all of the trees lie on the surface of a specific sphere, the best MDS solutions are circular. Also the MDS clustering, like the cartographic projection, has a large concentration of good trees. Unlike the cartographic projection of these 8 taxon sets (result not shown), however, the MDS formed two separate clusters and also has a scattering of good trees throughout a large portion of the visualization. Although it is not clear that the clustering of scores caused by MDS is inferior to that of cartographic projections, it is crucial to note that MDS is a post process step and cannot be used to guide a search. Therefore any structure is inherently not an exploitable structure.

### Results from Large Data Set Searches

It is not practical to exhaustively search the tree space associated with a large data set. Instead the phylogenetic search program PSODA [29] was modified to output every tree that it was going to perform a TBR rearrangement on, and every 100th rearrangement so produced. This gives not only the path of best trees found by the search as it progressed, but also a sampling of the trees that were rejected.

**Figure 9:**
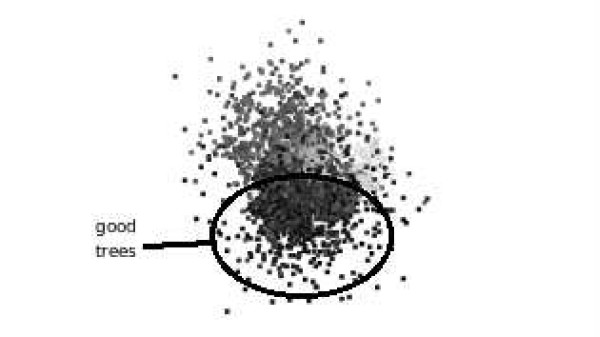
**Projection of a search through the Zilla 500 taxa data set**.

Figure 9 shows a projection of a TBR search with the Zilla data set [30] using cartographic projections. Again, a clustering of scores is apparent among the trees considered by the search, revealing exploitable structure in this difficult data set.

## Future Work

The cartographic projection from the hypersphere of trees has revealed significant structure to the problem of phylogenetic search. Further contributions can be made in improving our understanding of the revealed structure. More importantly new search techniques can be developed that can exploit this structure.

### Axis Optimization

The current projection from split space to the 3-D visualization is based on the random selection of the points in split space. These points are guaranteed to result in linearly independent reference vectors and are very likely to result in vectors which are orthonormal as well. Although the initial random selection provides encouraging results, a more intelligent selection of basis vectors could improve the quality of the visualization.

### Improved Phylogenetic Searches

There are two directions in which to take this work with respect to improving phylogenetic searches by utilizing the structure seen in the visualization. The first is to create a human guided search. As the projection from split space to the visualization is a simple linear transformation, it is possible to select a point in the visualized space and calculate the subspace of split space that corresponds to that point. A tree or trees in that subspace would then be generated and added to the list of trees used in a typical TBR based search, thereby restarting the search from the desired location. The second approach is to calculate and directly use the apparent gradient seen in the visualization to find better trees.

## Conclusions

This cartographic projection from  fulfills all defined criteria for a good visualization. First the mapping from *n*-trees to  is one-to-one and further the cartographic projection for  to ℝ^3 ^is linear. This means that each tree maps to exactly one point, and this point is not affected by any outside influences. Also because the mapping is linear, it is reversible, which meets the second criterion. Euclidean distance in  is easy to calculate. Robinson-Foulds distance is also closely related to  as both definitions are based on the partition sets of trees. Either of these distance metrics are easily calculated and meet our third criterion.

More importantly, the use of a cartographic inspired projection has revealed significant structure to the problem of phylogenetic search. The visualization shows a general clustering of trees with similar scores, and in some data sets a clear gradient structure is observed. This promises to be useful in furthering our understanding of the problem of phylogenetic search and for informing the development of new methods in the field. These new methods will expand our ability to perform phylogenetic analysis which has implications for many biological fields.

## Methods

The extremely high dimensionality of  makes explicit storage of the three reference vectors needed for the cartographic projection infeasible. Likewise, due to the size of these vectors the typical calculations used for computing inner products require infeasible amounts of time. A naïve implementation of cartographic projections is adequate for very small numbers of taxa, but more sophisticated techniques are required for most data sets.

## Hash Table Vector Representations

The memory usage of a straightforward implementation of cartographic projections is exponential in the number of taxa. Rather than explicitly storing the very large reference vectors a hash table representation is chosen. This representation has a fixed memory size, which can be arbitrarily chosen independently of the number of taxa. A similar hash table was used by Pattengale *et al*. [31] to quickly compute RF distances. Many of the assumptions necessary for computing RF distances, such as a small incidence of collisions, are violated in this work. However, the similarities do help to explain why the method of cartographic projections does so well at preserving RF distances.

To construct this table, a hash function and three representative vectors of a feasible dimensionality, one for each reference vector, are chosen. The hash function chosen must have a range equal to the set of nonnegative integers up to the dimensionality of the reference vectors and a domain equal to the set of natural numbers up to the dimensionality of the representative vectors.

Together these representative vectors and the hash function are used to compute the elements of the reference vectors as needed. The *i*^*th *^element of each reference vector is defined to be the element of the corresponding representative vector with the hashed value of *i *as follows:(1)

This representation allows a fixed amount of memory to be adequate for data sets of any number of taxa. This bound on memory usage is critical for the visualization of large data sets.

## Orthogonality and Normalization of the Reference Vectors

It is desirable that the three reference vectors be orthogonal to each other and also that they be normalized, so that we have an orthonormal basis for visualization. As the dimension of the three reference vectors is very large it is not practical to directly enforce either of these constraints. An additional complication is that each reference vector is not explicitly stored, but is instead implicitly defined by its representative vector and the hash function. Yet, with these constraints it is still possible to make the reference vectors mutually linearly independent and give bounds on their normality and orthogonality. These bounds and their proofs are given later in this section.

If the representative vectors are made to be orthogonal then regardless of the choice of hash function, the true reference vectors are linearly independent by Theorem 9. The quality of the orthogonality property of the reference vectors is dependent on the quality of the hash function as shown in Theorem 10. Given the size of the representative vectors used (65,535 elements) and only 20 taxa the angle between reference vectors is 90 ± 7.32 × 10^-5 ^degrees, very close to orthogonal. As the number of taxa increases this bound becomes even tighter.

Normalizing the reference vectors is more difficult. Due to the finite precision arithmetic of computers, it is not possible to normalize the reference vectors to unit length. As the vectors have a very high dimensionality, normalization tends to make each individual element too small to be represented, which in turn results in all of the reference vectors becoming the zero vector. As an alternative, each representative vector is made to have the same length as the others, without constraining this length to be one.

Again the normalization can only be performed on the representative vectors in the hash table. However Theorem 11 shows that the reference vectors are also normal if the hash function is perfectly even and gives a bound on how far off of normal the vectors can be in every other case.

The bounds given do not depend on the hash function, so any good hash function should be adequate. Bob Jenkins' *one-at-a-time *hash function [32] was used for the results in this paper.

## Calculating the Inner Product

The naïve method of calculating an inner product grows linearly with the dimension of the two vectors involved. Unfortunately, in this case the size of those vectors grows as the combinations of taxa. This method therefore gives worse than exponential performance with respect to number of taxa. However, for any given tree of *n *taxa, the vector representing that tree will have exactly *n *- 3 non-zero components by Lemma 3. Furthermore, each of these will be exactly one by the definition of trees in . These two properties can be exploited to give an algorithm that computes the needed inner products in time *O*(*n*), where *n *is the number of taxa.

This method begins with a hash table. Each element of the hash table contains one element from each of the reference vectors. The keys into the hash table are partition sets. The mapping of a tree is accomplished with the following steps.

• A list of the *n *- 3 partition sets is built: *O*(*n*)

• Each partition is used to lookup a set of *x*, *y*, and *z *values in the hash table: *O*(1) * *O*(*n*)

• The *n *- 3 values are summed giving the final mapping: *O*(*n*)

These steps give an overall runtime execution of *O*(*n*).

**Figure 10:**
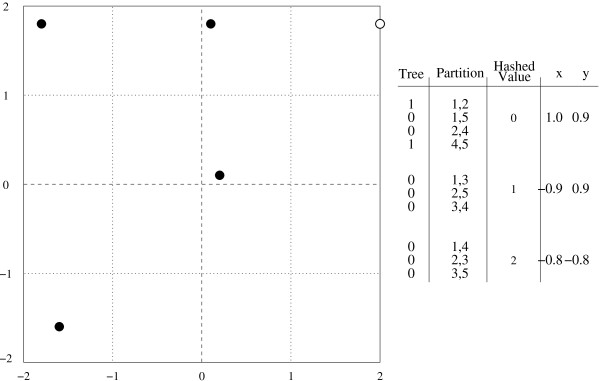
**A 2-D cartographic projection of all 5 taxon trees, with reference vectors represented through a simple modulo 3 hash**. Under this simple hash function and small representative table size there are only 5 locations which correspond to valid trees. This projection is therefore not one-to-one. In practice this possibility is mitigated by using a larger table. The vector for the tree ((1,2),3,(4,5)) is also shown. The point corresponding to this tree is highlighted in the graph.

For example, consider all trees of five taxa numbered 1-5 respectively. Every non-trivial branch has two taxa on one side and three on the other. The hash function will be computed as follows; add the taxon numbers of the two taxa on one side, then divide this sum by three and take the remainder as the value of the hash function. There are three possible values for this hash function 0, 1, and 2. Figure 10 shows the full reference vectors. A reference vector is assigned to each of these values. The reference vectors will be axis-aligned unit vectors, the value of 0 will correspond to the vector (1.0,0.9), 1 to the vector (-0.9,0.9) and 2 to the vector (-0.8,-0.8).

This scheme gives six possible locations for each of the fifteen possible trees to map onto, one of which does not correspond to any valid trees. These locations are all shown in Figure 10. In this example the tree ((1,2),3,(4,5)) maps to the point (2.0,1.8). The partition (1,2) as well as the partition (4,5) both map to the vector (1.0,0.9), these results are added together to obtain the final location of the tree.

This method has two main advantages. First the time needed to compute the inner product scales with the number of taxa rather than with the dimensionality of split space. Secondly only a fixed amount of storage for the hash table is required, regardless of the number of taxa in the tree. This upper bound on necessary storage makes the visualization of larger data sets feasible.

## Proofs

As givens in all of the following proofs are two vectors *X *and *Y*, each of dimension *d*. These vectors are arbitrarily chosen orthogonal vectors. They are also used to construct two vectors *X*' and *Y*', each of dimension *d*', using a hash function *h*.

This paper used three vectors of dimension 65535, with elements chosen randomly with a uniform distribution from [-1,1]. Using the Gram-Schmidt method these vectors were all made to be orthogonal to each other. Finally they were each modified to make their magnitudes equal to the magnitude of the first vector.

### Definitions

**Definition 5. ***h *is a function with the following properties:(2)

Such a function is easily constructed. One such function when *d *<*d*' is *h*(*x*) = *x *mod *d*.

**Definition 6. ***X*' and *Y*' are two vectors constructed from *X*, *Y*, and *h *as follows:(3)

**Definition 7. **The frequency with which *h *maps any number *j *onto a given number *i *is(4)

Note that *f*_*i *_has the following bounds:(5)

**Definition 8. ***ξ*, the quality of the function *h*, is a measure of how evenly the elements of *X*' and *Y*' are mapped by *h *onto *X *and *Y*.(6)

Note that due to the bounds on all *f*_*i*_, *ξ *has the following bounds:(7)

### Theorems

**Theorem 9. ***Given two orthogonal vectors X and Y, two arbitrarily larger vectors X' and Y' can be constructed such that they are linearly independent.*

*Proof*. As *X *and *Y *are orthogonal, they are also linearly independent. That is to say:(8)

Thus all equations of the form(9)

can be rewritten as(10)

In this fashion(11)

From which it is clear that *X*' and *Y*' are linearly independent.

**Theorem 10. ***Given two orthogonal vectors X and Y, two arbitrarily larger vectors X' and Y' can be constructed such that they are orthogonal within a given bound.*

*Proof*. Using Definition 7 the inner product ⟨*X*'|*Y*'⟩ can be written in terms of *X *and *Y*.(12)

As *X *and *Y *are orthogonal, their inner product ⟨*X*|*Y*⟩ = 0; therefore either(13)

and clearly(14)

or(15)

In this second case it may not be true that *X*' and *Y*' are orthogonal. Even so there are bounds on ⟨*X*'|*Y*'⟩. The largest possible magnitude of ⟨*X*'|*Y*'⟩ occurs when *h *maps each member of {ℕ <*d*} to one member of {ℕ <*d*'} with the exception of one element of {ℕ <*d*} which maps to the remaining elements of {ℕ <*d*'}. Furthermore, that sole exception corresponds with the largest magnitude of *X*_*i*_*Y*_*i*_. In this case the inner product is given by(16)

The angle *θ *between *X*' and *Y*' is given by(17)

Applying the bound on ⟨*X*'|*Y*'⟩, and the bounds on the magnitudes of *X*' and *Y*' from Theorem 11(18)

As *X *and *Y *are arbitrary but constant expressions, note that(19)

Therefore as the number of taxa increases the vectors in question approach orthogonality.

**Theorem 11. ***Given two vectors of equal magnitude X and Y, two arbitrarily larger vectors X' and Y' can be constructed such that they are also of equal magnitude within a given bound.*

*Proof*. As *X *and *Y *are of equal magnitude it is the case that(20)

The magnitude of *X*' is bounded above by(21)

As the range of *h *is in {ℕ <*d*} every element of *X *is also an element of *X*'. Therefore(22)

The magnitude of *Y*' is bounded in the same fashion. The ratio of the two magnitudes is bounded as follows(23)

Additionally, if *ξ *= 0 then(24)

and the two vectors have equal magnitude

## Competing interests

The authors declare that they have no competing interests.

## Authors' contributions

KS developed and implemented the methods. QS and MC both acted in an advisory role. All authors read and approved the final manuscript.
